# Postfire biodiversity database for eastern Iberia

**DOI:** 10.1038/s41597-023-02794-9

**Published:** 2023-12-06

**Authors:** Juli G. Pausas, Lola Álvarez-Ruiz, Arturo Baz, Josabel Belliure, Guille Benítez, P. Pablo Ferrer-Gallego, Salvador Herrando-Pérez, Joan Nicolau Jiménez, Emilio Laguna, Eduardo Mínguez, Sergio Montagud, Raimundo Outerelo, Vicente Roca, Xavier Santos, Antonio J. Velázquez de Castro, Amador Viñolas, Julio Cifuentes, José D. Gilgado

**Affiliations:** 1 Centro de Investigaciones sobre Desertificación (CIDE-CSIC), 46113 Valencia, Moncada Spain; 2 Department of Life Sciences, University of Alcalá, Madrid, Spain; 3 Direcció General del Medi Natural i Avaluació Ambiental, Generalitat Valenciana, 46018 Valencia, Spain; 4 BioCore S. Coop., Calle de Manzanares 4, 28005 Madrid, Spain; 5 Museu [UV] Història Natural, Universitat de València, Avinguda Dr. Moliner, 50, 46100 Valencia, Burjassot Spain; 6 Departamento de Biodiversidad, Ecología y Evolución, Facultad de CC. Biológicas, Universidad Complutense de Madrid, 28040 Madrid, Spain; 7 Departament de Zoologia, Facultat de Ciències Biològiques, Universitat de València, 46100 València, Burjassot Spain; 8 CIBIO/InBIO, Universidade do Porto, 4485-661 Vairão, Portugal; 9 Museu de Ciències Naturals de Barcelona. Laboratori de Natura. Coŀlecció d’Artròpodes. Passeig Picasso, s/n, E-08003 Barcelona, Spain; 10 Departamento de Biología, Facultad de Ciencias, Universidad Autónoma de Madrid, 28049 Madrid, Cantoblanco Spain

**Keywords:** Biodiversity, Fire ecology

## Abstract

In the summer of 2012, two fires affected Mediterranean ecosystems in the eastern Iberian Peninsula. The size of these fires was at the extreme of the historical variability (megafires). Animals are traditionally assumed to recolonize from source populations outside of the burned area (exogenous regeneration) while plants recover from endogenous regeneration (resprouting and seeding). However, there is increasing evidence of *in situ* fire survival in animals. To evaluate the effect of large-scale fires on biodiversity and the mechanism of recovery, in 2013, we set up 12 plots per fire, covering burned vegetation at different distances from the fire perimeter and unburned vegetation. In each plot, we followed the postfire recovery of arthropods, reptiles (including some of their parasites), and plants for 2 to 5 years. Here we present the resulting database (POSTDIV) of taxon abundance. POSTDIV totals 19,906 records for 457 arthropod taxa (113,681 individuals), 12 reptile taxa (503 individuals), 4 reptile parasites (234 individuals), and 518 plant taxa (cover-abundance). We provide examples in the R language to query the database.

## Background & Summary

Wildfires are natural processes in many ecosystems worldwide, especially in those experiencing highly seasonal climates; this includes some biodiversity hotspots. In recent years, we have learned a great deal about how plants respond to fire^1^. Albeit rapidly growing, our understanding of animal responses is more limited^2^. This is in part because terrestrial animals are highly diverse and mobile, so their responses to wildfires are often behavioural and difficult to detect. In comparison, plants are sessile and less diverse^3^ so their response to wildfires is typically morphological and easier to study. In Mediterranean ecosystems, plant populations have strategies to survive or to regenerate after a fire (endogenous regeneration mechanisms^1^) while animals are often considered to recolonise burned areas mainly from adjacent populations^4^. However, there is growing evidence for postfire survival in many taxa^5–12^. If recolonization is the main mechanism for animals to recover postfire, then animals should be more sensitive to fire size than plants; otherwise, other fire regime parameters may be more relevant (e.g., fire intensity). Thus, large fires are an opportunity to understand the effect of fire on biodiversity and the mechanisms driving the recovery.

**Fig. 1:**
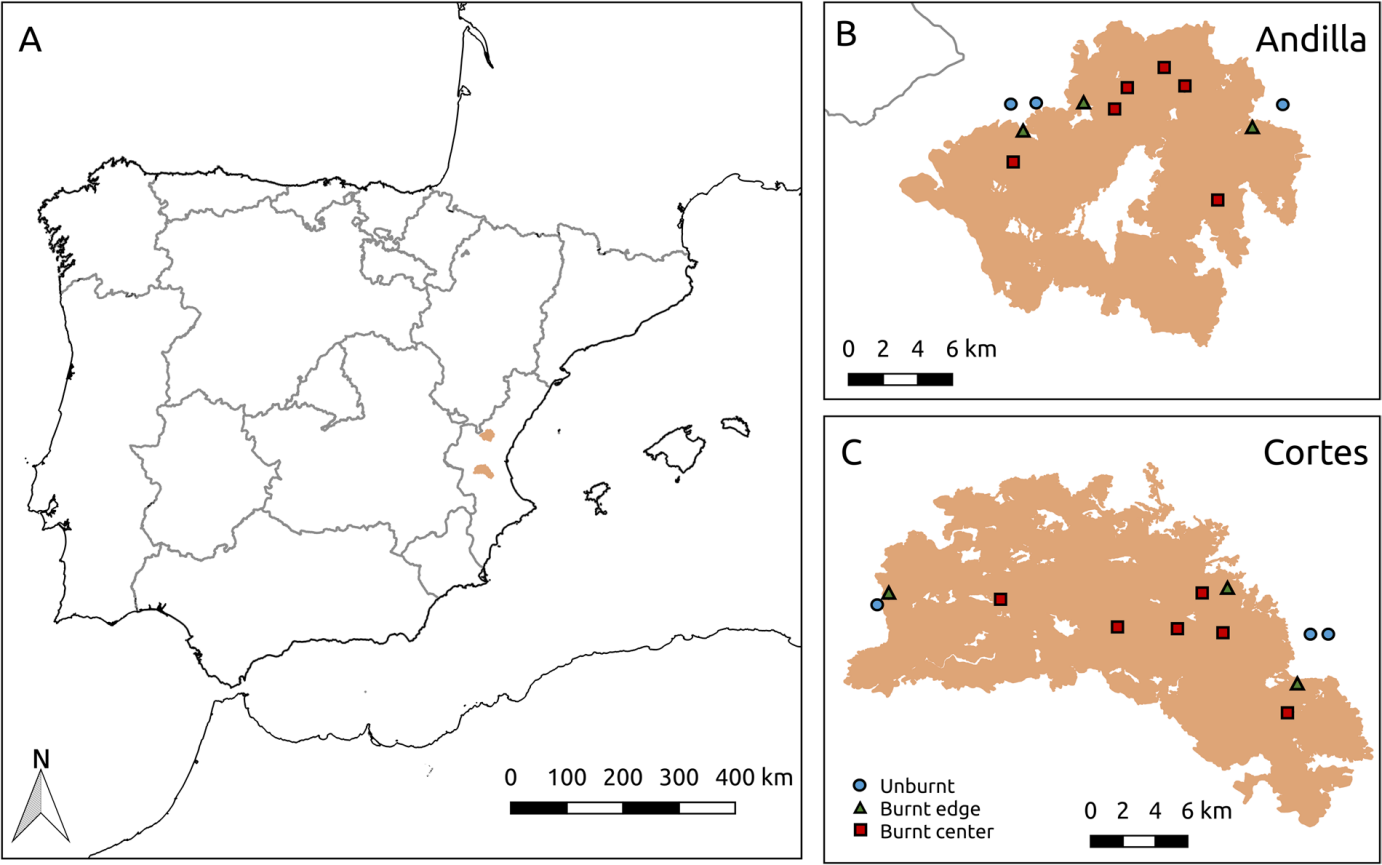
Location of the two study areas affected by the 2012 megafires in Andilla and Cortes (Valencia, Spain, eastern Iberian Peninsula). (**A**) Burned area (in orange) in Andilla (north) and Cortes (south). (**B**) Andilla burned area (~21,000 ha). (**C**) Cortes burned area (~30,000 ha; Table 1). In B and C, symbols represent sampling plots (circles = unburned; triangles = burned edge; red squares = burned middle and center), and unburned patches in white are villages and agricultural fields without natural vegetation. Modified from Pausas *et al*.^16^.

In 2012, two large fires (>20,000 ha) occurred simultaneously in the Valencia region (Spain, eastern Iberia; Fig. 1). Local records (19th and 20th centuries^13^) indicate that fires of this size are outliers and thus they can be considered *megafires*^14^. Fire size, intensity and history of these two simultaneous and neighbouring fires provide an excellent template to study the responses of plant and animal communities to wildfires. Here we present the POSTDIV database collating species and abundance of arthropods (2 to 4 years), lizards (and their parasites; 4 years) and plants (5 years) that were annually monitored during the regeneration process at different distances from the centre of each fire, plus in adjacent unburned areas.

## Methods

### The fires

**Fig. 2:**
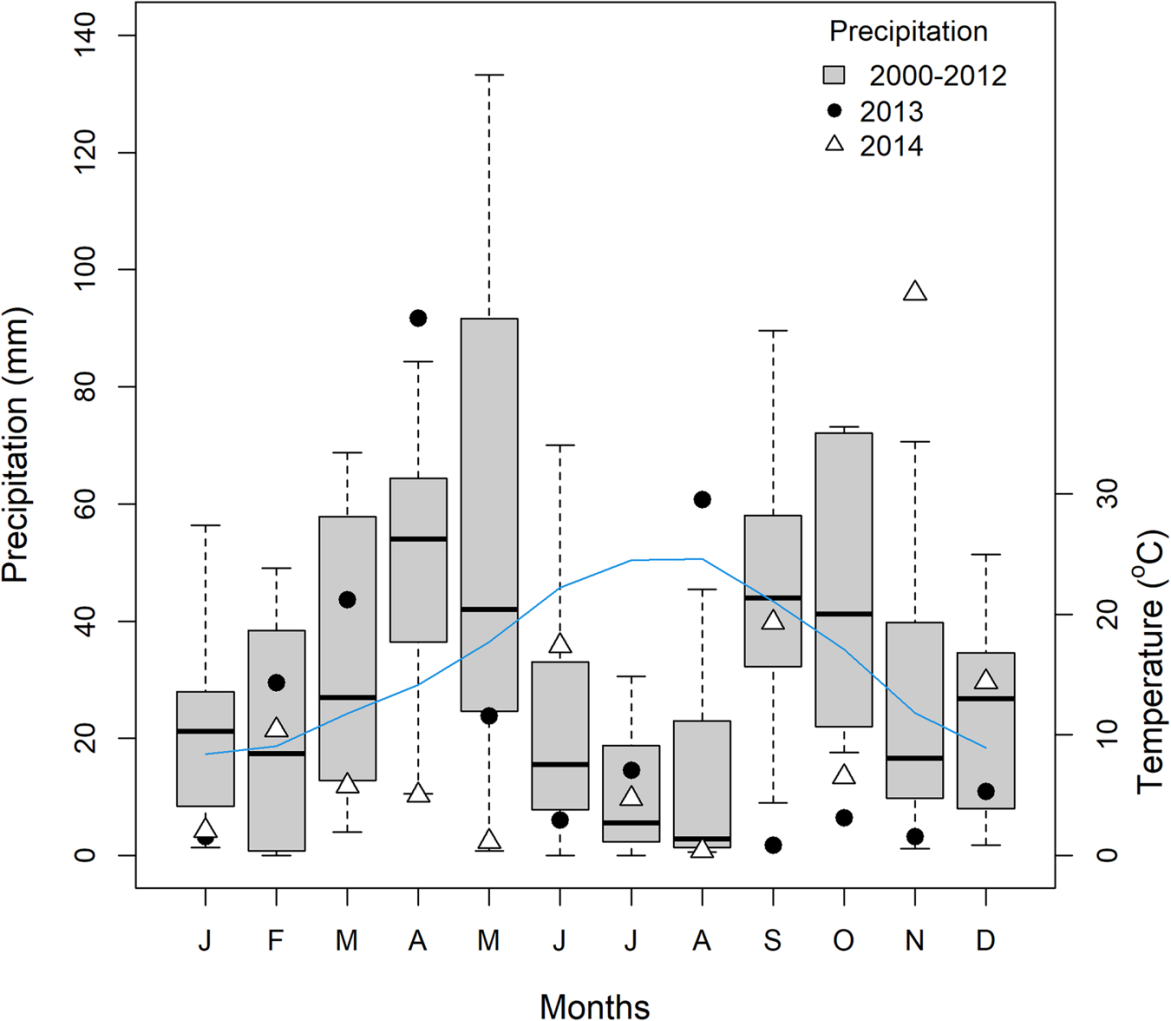
Climate variation recorded at the Llíria meteorological station (250 m asl, 39°39′50″N/0°39′14″W; owned by IVIA, http://riegos.ivia.es) located between the 2012 Andilla and Cortes fires (Valencia, Spain, eastern Iberian Peninsula). Boxplots show monthly variability in precipitation from 2000 to 2012 (left axis). Symbols represent monthly precipitation in 2013 (black circles) and 2014 (white triangles). Blue line shows mean monthly temperature (°C, right axis) from 2000 to 2012 (mean daily temperature averaged by month and year). Interannual variability in temperature (not shown) was much lower than in precipitation. Source: Pausas *et al*.^16^.

The two fires occurred simultaneously in June-July 2012 in the Valencia region (Fig. 1, Table 1). The area has a Mediterranean climate (Fig. 2) and a long history of fire activity^13,15^. The fires burned under extreme conditions (hot dry weather with strong winds), starting in the municipalities of Andilla and Cortes de Pallás (hereafter, Andilla and Cortes fires; Fig. 1, Table 1). They burned at high intensity, avoiding only villages, farms, and agricultural fields. Burning mostly affected entire plants (crown-fires), except in some margins (excluded from sampling) of the Andilla fire. Before 2012, much of the Cortes study area was a shrubland dominated by woody species (mostly *Quercus coccifera, Rhamnus lycioides, R. alaternus, Phillyrea angustifolia, Pistacia lentiscus, Juniperus oxycedrus, Cistus albidus, C. clusii, C. monspeliensis, Rosmarinus officinalis*), with some important herbaceous species (e.g., *Macrochloa tenacissima* and *Brachypodium retusum*). The Andilla study area alternated similar shrublands with pine woodlands (*Pinus halepensis*) and patches of evergreen oak (*Quercus ilex rotundifolia*).

**Table 1:** General characteristics of the two fires considered in the POSTDIV database.

	Andilla fire	Cortes fire
Municipality of initiation	Andilla	Cortes de Pallás
Fire size (ha)^a^	20,945	29,752
Elevation (m; mean and range)	952 (600–1200)	441 (190–750)
Distance between plots (km; range)	1.4–16.0	1.0–28.0
Maximum plot distance to fire perimeter (km)	2.8	3.0
Number of previous fires (1975–2011)	0	1–4

^a^Total fire size including unburned patches amount to 23,334 (Andilla) and 31,450 (Cortes) ha.

The two fires were 65 km apart (straight line) in different mountain ranges separated by an agricultural valley (where the Llíria meteorological station is located; Fig. 2). The Cortes and the Andilla study areas had their own pre-2012 fire history. Cortes had experienced recurrent wildfires (1978 to 1994), particularly in the center of the area burned in 2012. This is expected because, even if fires start near the mountain range’s margin, the core of a mountain range, covered by natural vegetation and surrounded by farmland, is likely to burn more frequently than the periphery. Consequently, distance from the 2012 fire perimeter is correlated with fire history in Cortes. In contrast, no wildfires had occurred in Andilla in the period 1978–2011.

### Plots

We sampled a total of 24 plots, 12 per site (Andilla and Cortes fires). For each site, we selected three plots in the surrounding unburned area (‘Unburned zone’) and three sets of three plots at each of three distances from the fire perimeter in the burned area (<700 m: ‘Edge zone’; ~1.5 km: ‘Middle zone’; >2 km: ‘Center zone’). Middle and Center plots were relatively close to each other, and based on beetle responses to the two fires, the two zones were considered a single category (‘Center zone’) in Pausas *et al*.^16^. The POSTDIV database provides the two plot classifications (Zone4 and Zone3; see below Table 2), as well as the specific distance (km) to fire perimeter for each plot (Table 2).

**Table 2:** Plot descriptors in the database POSTDIV.

Column name	Descriptor
Plot	Unique identifier per plot (24 unique strings, Table 3)
Site	Andilla or Cortes (Table 1): the municipality where fires started
Municipality	Municipality of the plot (fires affected multiple municipalities)
Latitude	Geographical coordinates (decimal degrees, WGS84)
Longitude	Geographical coordinates (decimal degrees, WGS84)
Altitude	Elevation above sea level (m)
Dist2border	Distance to the perimeter of the fire (straight line, km)
Zone3	Plot assignment to three zones (categorical: Unburned, Edge, Center)^16^
Zone4	Plot assignment to four zones (categorical: Unburned, Edge, Middle, Center)^11^
Fire.rec	Fire recurrence (number of fires from 1978 to 2011)
Bare	Proportion of bare soil in spring 2013 (%)
Stones	Proportion of stones in spring 2013 (%)
Herbs	Herb cover in spring 2013 (%)
Woody	Wood cover in spring 2013 (%)
Hherbs	Herb height in spring 2013 (cm)
Hwoody	Wood height in spring 2013 (cm)

Because of the large size of the fires, the distance between plots (within each fire) was considerable (Table 1) and plots were often located in different watersheds. Plots in the burned area (Edge, Middle and Center zones) were located in shrublands totally affected by crown-fires; we deliberately avoided 2012-prefire densely forested areas from sampling. Plots in the Unburned zone consisted of mature shrublands outside of the fire perimeter. The Andilla fire occurred at higher altitude than the Cortes fire, hence Andilla plots were on average ~500 m above Cortes plots (Table 1). Importantly, the 2014 spring was much drier than the 2013 spring, and drier than springs in most previous years (Fig. 2). Overall, our sampling design accounted for prevailing environmental variability found in the shrublands of the study region.

### Arthropods

At each plot, we placed four pitfall traps at the corners of an imaginary 25 × 25 m sampling plot (48 traps per site: 4 traps/plot × 3 plots/distance × 4 distances). Traps were 1 L plastic cups (top inner diameter = 10 cm, depth = 15.5 cm) buried in the soil with the top at ground level, and covered with a tile a few centimeters above the soil. Traps were filled (~60% cup volume) with propylene glycol (Anorsa, Barcelona, Spain).

Pitfall traps were set in May 2013 and 2014 in both sites and in 2016 in Cortes only. Each year traps were collected roughly monthly between June and August. In 80% of the cases, the number of days between monthly samples (interval with trap activity) ranged between 21 and 29 days. Counts per taxon were recorded by plot after pooling counts from the four traps. As some of the traps were defective (e.g., removed by large ungulates), POSTDIV includes the number of active traps and the sampling dates per plot, so users can easily standardize count data by sampling effort (see “Usage notes” and example 9 in section “Code availability”).

Pitfall fauna was sorted under a binocular microscope at the taxonomic level of Order. Then ants (family Formicidae), beetles (order Coleoptera), isopods (order Isopoda), millipedes (class Diplopoda), and spiders (order Araneae) were identified to the lowest taxonomic level possible. Count data for the beetle genus *Protaetia* have been analysed previously^16^.

### Reptiles

We searched for reptiles visually above ground, below rocks, and in shelters in the first four postfire years (2013 to 2016). Annual sampling was conducted in spring (April-June) on sunny days (air temperatures = 20–25 °C) when reptiles are most active and within their reproductive period. Each plot was surveyed three times (separated by at least one week) per year. Each search was done by two researchers for 30 minutes (sampling effort = 1 h). All plots were visited within a 4-5-day period in each sampling month and year. Specimens were identified to species level in the field. Count data for the most common lizards have been analyzed previously^11^.

### Lizard parasites

We collected faecal samples from lizard individuals (see Reptiles above) after an abdominal massage, and transported them to the lab for searching endoparasites.

Some lizards accidentally fell in the pitfall traps. Those lizards were fixed and stored in 70% ethanol in the field, then dissected in laboratory using a stereo-microscope. We collected all helminths found in the digestive system.

Parasites from both the digestive content and faecal samples were rinsed, fixed and mounted according to standard techniques^17^ and identified following Bush *et al*.^18^. We found nematodes, platyhelminths (Cestoda; Roca *et al*. 2020), and protozoans (Coccidia). Despite we were not able to identify the genus of the Coccidia due to the lack of sporulated oocysts, we provide their presence as this is uncommon in lizards^19^.

### Plants

In the first postfire year (2013) we visually estimated height and cover for woody and herbaceous vegetation in four places of each plot, in the vicinity of each pitfall trap. We then averaged height and cover per plot. These estimates were used to characterize vegetation structure at the plot scale in previous studies^16^. For each plot, we annually monitored (in spring) plant species during 5 years postfire (2013–2017). For each species, we assigned a cover-abundance rank (between 1 to 6) following the Braun-Blanquet ordinal scale^20^.

## Data Records

**Fig. 3:**
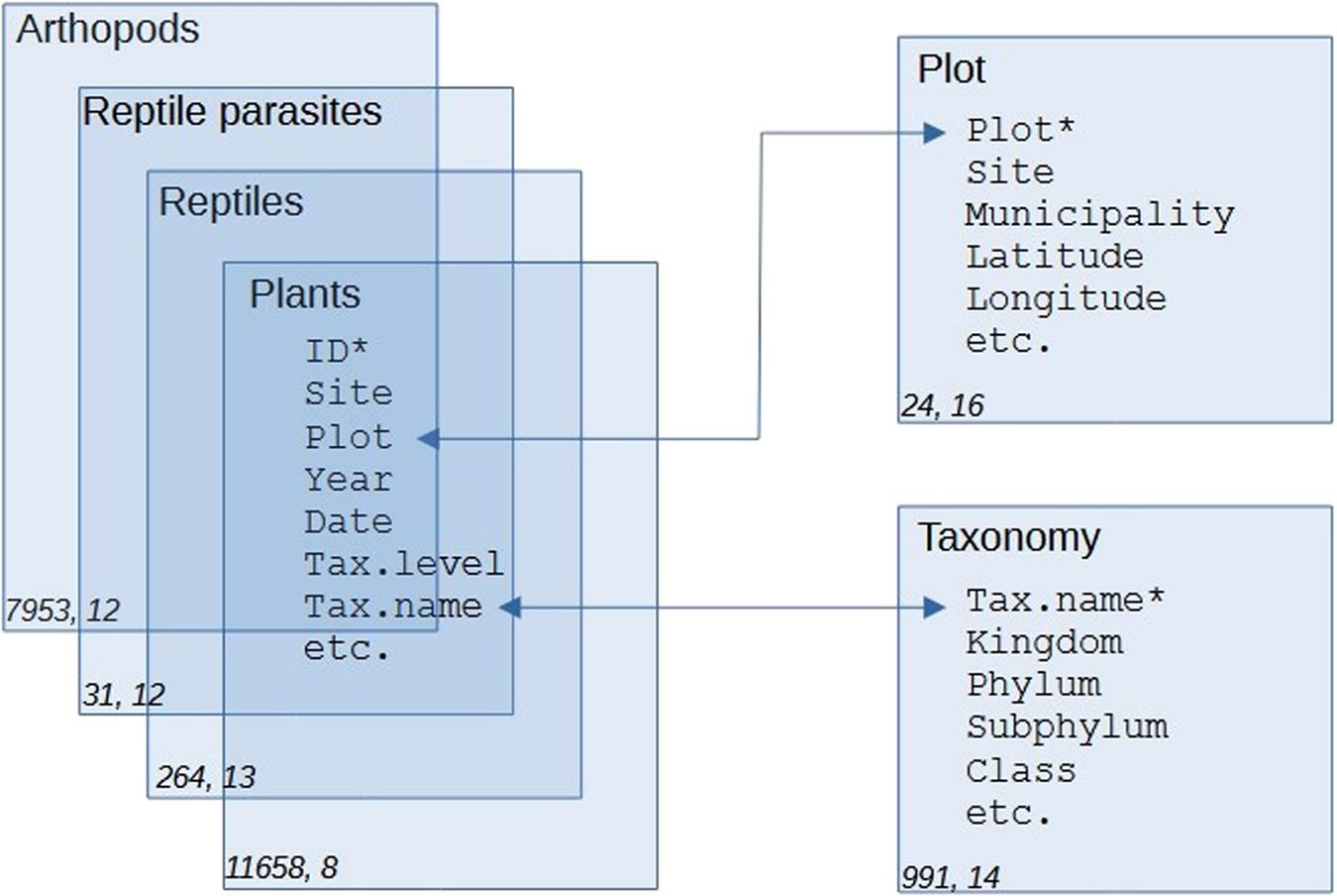
The POSTDIV database is composed of six data tables (blue boxes). Descriptors (fields) represent columns in the data tables (see Tables 2, 5 for a complete list of descriptors). Connector lines link data tables with taxon abundance (arthropods, reptiles, parasites, plants; left boxes) to data tables with taxonomic assignments and plot descriptors (right boxes). Asterisks denote unique identifiers in each data table. Numbers in the lower-left corners of the boxes represent number of rows and number of columns per data table.

The POSTDIV database^21^ consists of six data tables (Fig. 3) provided as a comma-separated text files (*.csv). Missing values are represented by empty cells; decimal places are dots. Four of the data tables include the main data (postdiv.arthopods.csv, postdiv.reptiles.csv, postdiv.parasites.csv, postdiv.plants.csv; for details see Tables 3, 4), and two additional data tables provide the plot descriptors (postdiv.plots.csv; Table 2) and taxonomic assignments (postdiv.taxonomy.csv; Table 5).

**Table 3:** Descriptors common to the four data tables containing taxon counts or abundance (arthropods, reptiles, parasites, plants) in the POSTDIV database.

Name	Descriptor
ID	Unique record identifier (3 letters + 4 numbers: XXX0000)
Site	Andilla or Cortes (Table 1); short name of the fire
Plot	Plot identifier (24 distinct codes with three characters) (Table 2)
Year	Sampling year
Date	Sampling date (YYYY-MM-DD)
Tax.level	Lowest taxonomic level identified
Tax.name	Taxonomic name (Table 5)

In those data tables, each row represents a single record, i.e., abundance of a given taxon per plot and date.

**Table 4:** Descriptors specific to each of the four data tables containing taxon abundance (Table 3; arthropods, reptiles, parasites, plants) in the POSTDIV database.

Column name	Descriptor
*Arthropods*	
N.individuals	Count of individuals (in pitfall traps) per taxon, plot and sampling date (see Table 3) (quantitative)
Sampling	Annual sampling-date order (from 1 to 4) for compatibility with Pausas *et al*.^16^
Ndays.traps	Number of days with pitfall activity (quantitative)
N.traps	Number of pitfalls providing count data per sampling date (quantitative: 1 to 4)
Details	Male (M); Female (F); Juvenile or Immature (J). For ants: queen (Q); worker (W); soldier (S) (categorical, *)
*Reptiles*	
N.individuals	Number of individuals (quantitative). Note that N.individuals ≥ N.adults + N.subadults as some individuals were not aged.
N.adults	Number of adult individuals, according to species-specific body sizes (*)
N.subadults	Number of individuals with body size smaller than that of adults, including juveniles (*)
Visit	Annual visit order (from 1 to 3) for compatibility with Santos *et al*.^11^ who summed the number of individuals from all three visits per year
Time	Start sampling time (hh:mm) in the day of sampling
Temperature	Start sampling air temperature (°C)
Clouds	Start sampling cloudiness (ordinal: 0 = clear to 6 = completely cloudy)
Wind	Start sampling wind speed (ordinal: 0 = no wind to 6 = strong wind)
*Lizard parasites*	
N.individuals	Count of individual parasites (quantitative)
Details	Male (M); Female (F); Egg (E); Larvae (L).
Host	Scientific name of the host lizard species
Host.sex	Sex of the host: male (M), female (F)
Where	Parasite location, 2 classes (faecal pellet, gut)
*Plants*	
Abundance	Cover-abundance index (ordinal: from 1 to 6) where 1: <5% cover & uncommon (few individuals); 2: <5% cover & common (many individuals); 3: 5–25% cover; 4: 26–50% cover; 5: 51–75% cover; and 6: 76–100% cover.

Asterisks (*) denote variables with missing values.

**Table 5:** Taxonomic descriptors in the POSTDIV database.

Column name	Descriptor
Tax.name	Unique identifier (see Table 3)
Kingdom	Taxonomic rank (Plantae, Animalia, Protista)
Phylum	Taxonomic rank
Subphylum	Taxonomic rank
Class	Taxonomist rank (for plants: Magnoliopsida and clades Monocots and Eudicots)
Subclass	Taxonomic rank
Order	Taxonomic rank
Suborder	Taxonomic rank
Family	Taxonomic rank
Subfamily	Taxonomic rank
Genus	Taxonomic rank
Subgenus	Taxonomic rank
Species	Binomial species name (equates with the Tax.name for records identified to species level)
NCBI	ID in the NCBI taxonomy database (National Center for Biotechnology Information) https://www.ncbi.nlm.nih.gov/Taxonomy/Browser/wwwtax.cgi; for animals only. For plants, we followed the taxonomic criteria by Mateo and collaborator^30,31^.

**Fig. 4:**
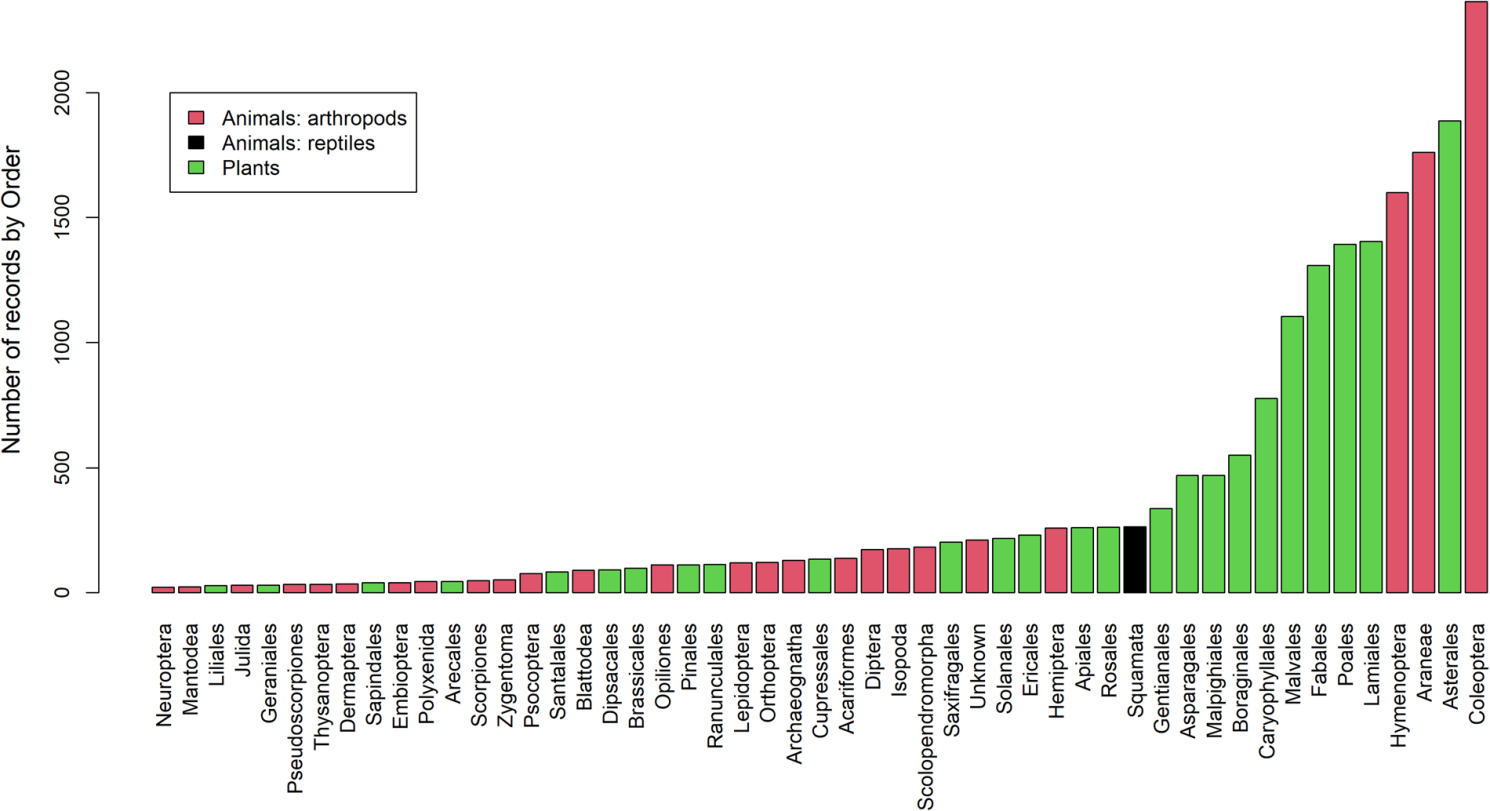
Number of records in the POSTDIV database (see Tables 3, 4) by Kingdom (colours) and Order (ordinate axis, only orders with >20 records).

**Fig. 5:**
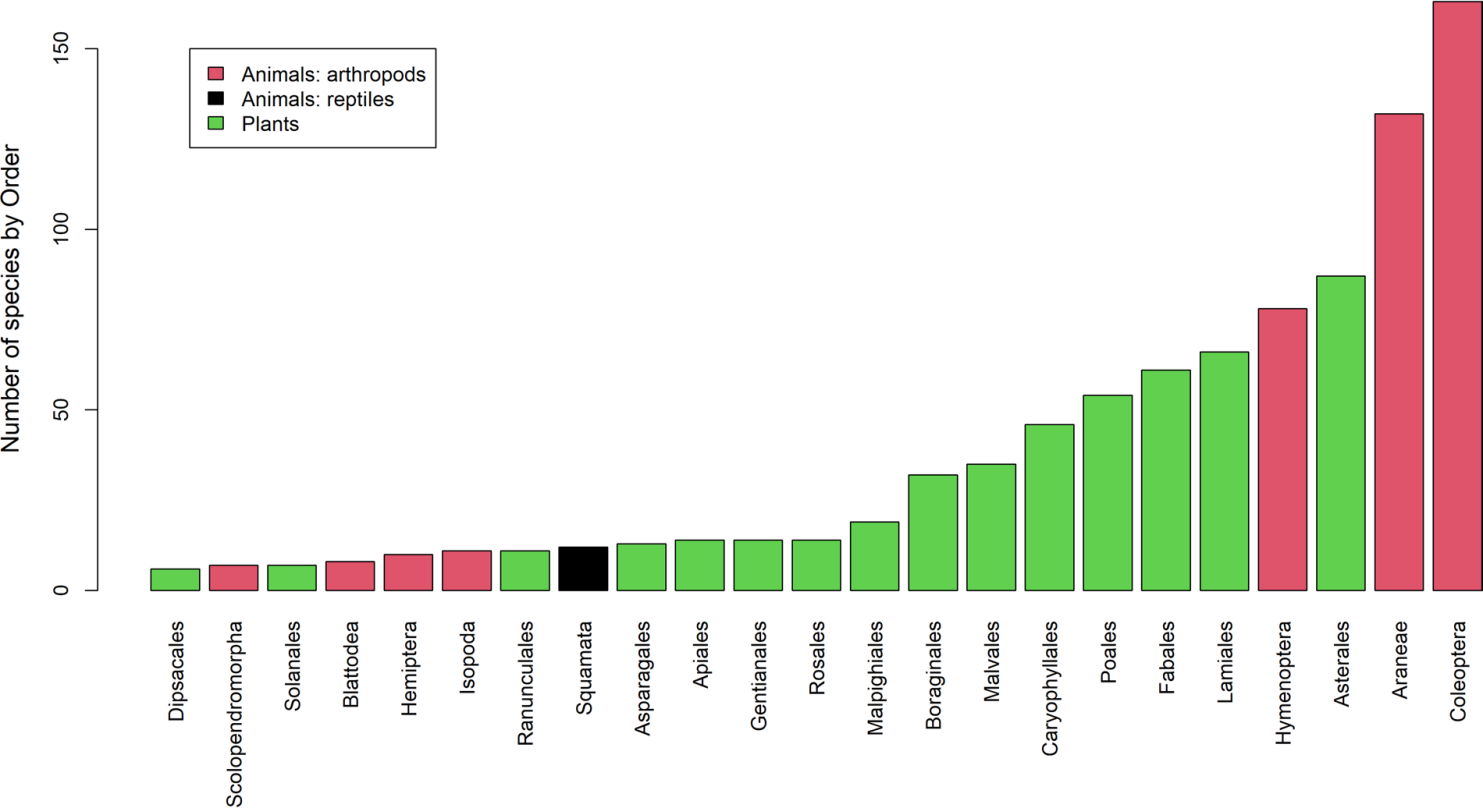
Number of species (arthropods, reptiles, plants) in the POSTDIV database by Kingdom (colours) and Order (ordinate axis, only orders with >5 species).

The database totals 19,906 records from 457 arthropods (113,681 individuals), 12 reptiles (501 individuals from 7 lizard and 5 snake species) and 518 plant taxa, along with 31 records from 4 parasitic taxa. Overall, the database includes taxa from 499 genera in 150 families and 66 orders. Araneae (spiders), Coleoptera (beetles) and Hymenoptera (ants, bees and wasps) outnumbered other arthropods taxa in abundance and species richness. The Algerian (*Psammodromus algirus*) and the East Iberian (*Psammodromus edwarsianus*) sand racers (Lacertidae lizards) dominated reptile observations. Asterales were the dominant plant order (Figs. 4, 5). Parasites include two nematodes, one cestode and coccidia. Most of the records (89%) correspond to organisms identified to species level; the remaining records are assigned at higher levels and may include multiple species. Further taxonomic work is currently in progress and will be updated in future versions of the database.

## Technical Validation

A subset of the database (*Protaetia* beetles, most lizards and their parasites) has been analysed and used in scientific publications^11,16,22^; a paper analysing arthropod communities is in progress. Those analyses allowed to find and correct errors. The final files of POSTDIV have been carefully screened for additional errors with the help of an R script generated for this purpose and available with the data (file “postdiv.check.R”; Data Citation 1).

Arthropod sampling effort varied across fires and sampling dates but can be easily standardized (see section “Usage notes”).

Reptile detectability can vary according to postfire vegetation structure^23,24^. Active search sampling of reptiles in other Mediterranean sites has however resulted in unbiased reptile-detection distance in unburnt versus burnt plots^25,26^. For this reason, we did not apply any correction to the reptile count data.

Arthropod taxonomy can be challenging. We linked each taxa identified to their unique identifier in the NCBI taxonomy database (National Center for Biotechnology Information, Table 5), except for taxa that have been recently described, often in specialized journals^27–29^. Plant taxonomy followed Mateo and collaborators^30,31^.

## Usage Notes

Arthropods count data is based on four pitfall traps *per* plot that were left active in the field for an average of 26 ± 4 days. However, in some cases, some pitfall traps were unavailable (mean number of traps = 3.9, sd = 0.31; e.g., removed by ungulates), and the number of days in the field varied among plots and sampling dates (min: 18, max: 34). Thus, for comparison, the count data (N.individuals) requires standardization by the sampling effort, that is, using the number of days the traps were in the field (Ndays.traps) and the number of traps considered in each sampling (N.traps; see Table 4). For instance, to standardize the count data to 4 traps in 30 days, the user can perform the following transformation (see also example 9 in the section “Code availability”):

(N.individuals/Ndays.traps/N.traps)*30*4

## Data Availability

The six data tables can be directly downloaded from Figshare^21^ and imported into any spreadsheet, database or statistical software. For instance, in R, the files can be simply uploaded as follows: art <- read.csv("postdiv.arthropods.csv") rep <- read.csv("postdiv.reptiles.csv") pla <- read.csv("postdiv.plants.csv") par <- read.csv("postdiv.parasites.csv") plot <- read.csv("postdiv.plots.csv") tax <- read.csv("postdiv.taxonomy.csv") We provide a series of examples of code in the R language to extract basic information from the database in the file “postdiv.examples.R”.
